# Priming with skin-problems increases fear of clusters

**DOI:** 10.1038/s41598-021-89917-7

**Published:** 2021-05-14

**Authors:** Risako Shirai, Hirokazu Ogawa

**Affiliations:** 1 Faculty of Science and Engineering, Waseda University, 3-4-1 Okubo, Shinjuku-ku, Tokyo, 169-8555 Japan; 2 Japan Society for the Promotion of Science, Tokyo, Japan; 3 Department of Integrated Psychological Sciences, Kwansei Gakuin University, Nishinomiya, Japan

**Keywords:** Psychology, Human behaviour

## Abstract

Trypophobia is induced by viewing multiple clustered objects. To date, several studies have investigated why certain people experience discomfort when looking at clustered patterns. Recently, “involuntary protection against dermatosis” (IPAD) hypothesis was proposed to explain the causes of trypophobia. The IPAD hypothesis suggests that involuntary aversive responses to skin diseases cause discomfort in response to image clusters. However, this idea has not been fully investigated empirically. Therefore, the present study used a modified version of the priming procedure and tested whether the activation of the concept of skin-related diseases affected the evaluation of trypophobic images. In Experiment 1, participants engaged in a lexical decision task in which words related to skin problems, negative events, or neutral events were presented. Then, they evaluated the discomfort of trypophobic, negative, and neutral images. The results indicated that participants evaluated trypophobic images as more discomforting after they were exposed to skin-problem-related words, whereas the exposure to words related to skin-problems did not enhance the discomfort of negative images. These findings demonstrate that the association with skin-related problems increases the discomfort of trypophobic images. In Experiment 2, we further tested the reproducibility of the priming effect observed in Experiment 1 and investigated the effect of priming with words related to COVID-19 in the context of a spreading infection. Contrary to predictions, no priming effect was produced by either skin-related words or COVID-19-related words. Future studies should further explore the causal relationship of the association between skin disease and trypophobia.

## Introduction

Trypophobia is the phobia for clusters of small holes or bumps, such as lotus seed heads, bubbles, and sea sponges. Many people have trypophobia in their daily life^1–4^ although it is not a specific DSM-V diagnosis. For example, some people feel uncontrollable fears when looking at sliced bread, Gruyère cheese, and clothes with dot prints^5^. On the other hand, it seems that others enjoy viewing trypophobic images.

Although several studies have discussed the causes of the discomfort experienced when viewing the trypophobic images, a consensus has not been reached on the causes of this phenomenon. To date, two major hypotheses regarding the causes of trypophobia have been proposed. The first is related to the spectral components of trypophobic images^1,6–11^. The log contrast energy is known to decrease linearly with increasing log spatial frequency *f* in natural images^12^. Some studies have suggested that images with deviation from a 1/*f* structure induce discomfort because the visual system has been tuned to process natural scenes efficiently (e.g., ref.^6^). Trypophobic images have high-contrast energy at midrange spatial frequencies, and this atypical power spectrum of trypophobic images might cause discomfort. Moreover, a recent study has indicated that phase spectrums of trypophobic images play a more significant role than the power spectrums in determining discomfort ^27^. Thus, relationships between spectral components of trypophobic images and trypophobic discomfort have drawn increasing attention.

The second hypothesis is based on cognitive appraisals, including disgust. It has been suggested that viewers feel disgusted by trypophobic images because their appearance is associated with diseases and infections^13–18^. Especially, Yamada and Sasaki^17^ proposed the “involuntary protection against dermatosis” (IPAD) hypothesis, which emphasizes the instinctive aversion to skin-related diseases as an explanation of the cause of trypophobia. According to the IPAD hypothesis, viewers involuntarily avoid trypophobic images because skin-related diseases cause visual patterns similar to trypophobic images resulting in the discomfort of these images. Indeed, empirical data have supported this hypothesis^14,17^. For example, Yamada and Sasaki^17^ demonstrated that the people that had suffered from skin disease experienced more discomfort of trypophobic images than those that had never suffered from skin diseases, which is suggestive of a relationship between skin disease and trypophobia. Therefore, previous studies suggest that the sensitivity to skin-problems might explain trypophobia. However, the causal mechanisms between high sensitivity to skin-problems and the discomfort for trypophobic images remain unclear because conclusions of previous studies have been limited to a correlation between the history of skin-disease and trypophobia.

In the present study, we manipulated the activation of skin-problem related concepts through a modified version of the priming method, which alters subsequent behaviors or judgments as a result of exposure to specific stimuli^19–21^. Then, we examined whether the association with skin-related problems enhances the discomfort for the trypophobic images. Participants were exposed to words related to skin-problems in a lexical decision task in which they reported whether the stimuli on the display were words or non-words. After that, the participants were presented various images, including trypophobic images, and their discomfort of the images was evaluated. We expected that if the concepts associated with skin diseases are activated when viewing the trypophobic images and which arises the discomfort for the trypophobic images, the pre-activation of skin-problem concepts through the priming would strengthen the link between the skin diseases concepts and trypophobia. Therefore, we predicted that the priming of skin-problems would induce stronger discomfort when viewing trypophobic images. Moreover, we predicted that the discomfort for trypophobic images would not increase when viewing negative words that do not activate the concept of the skin-problems.

## Experiment 1

### Methods

#### Participants

The sample size of the present study was determined as follows. Previous online studies have recruited about 100 participants per experimental group when performing the tasks of evaluation for the images (e.g., ref. ^3^). Thus, according to the previous study, 100 participants were set up for each experimental group to evaluate an image, resulting in the sample size of 903 (367 men and 536 women, mean age = 38.82 years, SD = 10.77). They were recruited from Crowd Works that is an online crowdsourcing service. All participants provided their informed consent through the Internet before participating in the study. Ethical approval for the study was obtained by the Kwansei Gakuin University Institutional Review Board for Behavioral Research with Human Participants, and all procedures were carried out in accordance with relevant guidelines and regulation.

### Stimuli

We used three types of word lists as stimuli; words related to skin diseases and injuries, emotionally negative words not related to the skin-problems, and emotionally neutral words. Moreover, the non-words were randomly generated from anagrams of Japanese characters (see S1). We conducted a preliminary survey to confirm the strengths of the valences of skin problem related and emotionally negative words. The participants (n = 630) responded to the preliminary survey online through CrowdWorks. They evaluated the discomfort related to 130 words (65 skin problem related words and 65 negative words unrelated to skin-problems) using an 11-point scale ranging between 0 (*Not unpleasant at all*) to 10: (*Extremely unpleasant*). We selected 40 words (20 skin-problem and 20 negative words) from the 130 words based on the rating scores for each word. The mean discomfort rating for the selected words related to skin-problems was 6.28 (SD = 2.53), and the mean discomfort rating for the selected words related to negative events was also 6.28 (SD = 2.50). We finally selected 20 skin-problem-related words, 20 negative words, and 20 neutral words (see S1).

The images consisted of 17 trypophobic, 17 negative, and 17 neutral images. The trypophobic images were selected from the Internet site, https://www.trypophobia.com, which exhibits images that are frequently associated with trypophobia such as lotus seed heads and honeycombs. The negative images contained emotionally negative objects such as guns and barking dogs, and neutral images were selected from the International Affective Picture System (IAPS^22^; see S2) and the Open Affective Standardized Image Set (OASIS^23^; see S2). All images were altered to grayscale and resized to 256 pixels × 256 pixels in MATLAB R2015a (Mathworks, Natick, MA).

### Procedures

The experiment consisted of an exposure session and an evaluation session, which was implemented in a browser window using the Qualtrics platform (Qualtrics, Provo, UT). Participants engaged in a lexical decision task in the exposure session. Each trial started with the presentation of a word or non-word. Then, after 3000 ms, two buttons (i.e., “word” and “non-word”) appeared on the display. The participants were instructed to press the “word” or the “non-word” button on the display by using the computer mouse according to their decision as quickly and accurately as possible. Each trial ended when the participants responded. The lexical decision task included 40 trials. The participants were randomly assigned to one of three groups and presented with different word stimuli in the exposure session. The three groups consisted of the group exposed to words about skin-problem (n = 306, 129 men and 177 women, mean age = 39.25 years; skin-problem word group), the group exposed to negative words not related to skin-problem (n = 303, 129 men and 174 women, mean age = 39.22 years; negative word group), and the group exposed to emotionally neutral words (n = 294, 109 men and 185 women, mean age = 37.95 years; neutral word group). The identical non-words were used with all three groups.

The evaluation session started after participants completed the exposure session. Seventeen images were randomly selected from 51 images (17 trypophobic, 17 negative, and 17 neutral images), such that 5–6 images were selected from each image type. The participants were instructed as follows: “Please rate the strength of the discomfort you feel when viewing the image by clicking a number on the 11-point scale ranging from 0 (*Not unpleasant at all*) to 10 (*Extremely unpleasant*).” Each image was presented on the display until a response was made. The evaluation session consisted of 17 trials.

We used a mixed design with the exposed word type (skin-problem, negative, and neutral) as a between-participant factor and image type (trypophobic, negative, and neutral images) as a within-participant factor. The discomfort score was subjected to a linear mixed model analysis with word and image type and their interaction terms using REML, a method for estimating the variances in models with random effects. We treated the participants as a random variable. The word and image types were coded by contrast coding (word type: reference = “neutral word”; image type: reference = “neutral image”). All analyses were conducted using the free statistical software R (version 3.5.1^24^). The linear mixed model was conducted by using lme4 packages^25^.

### Results and discussion

**Figure 1:**
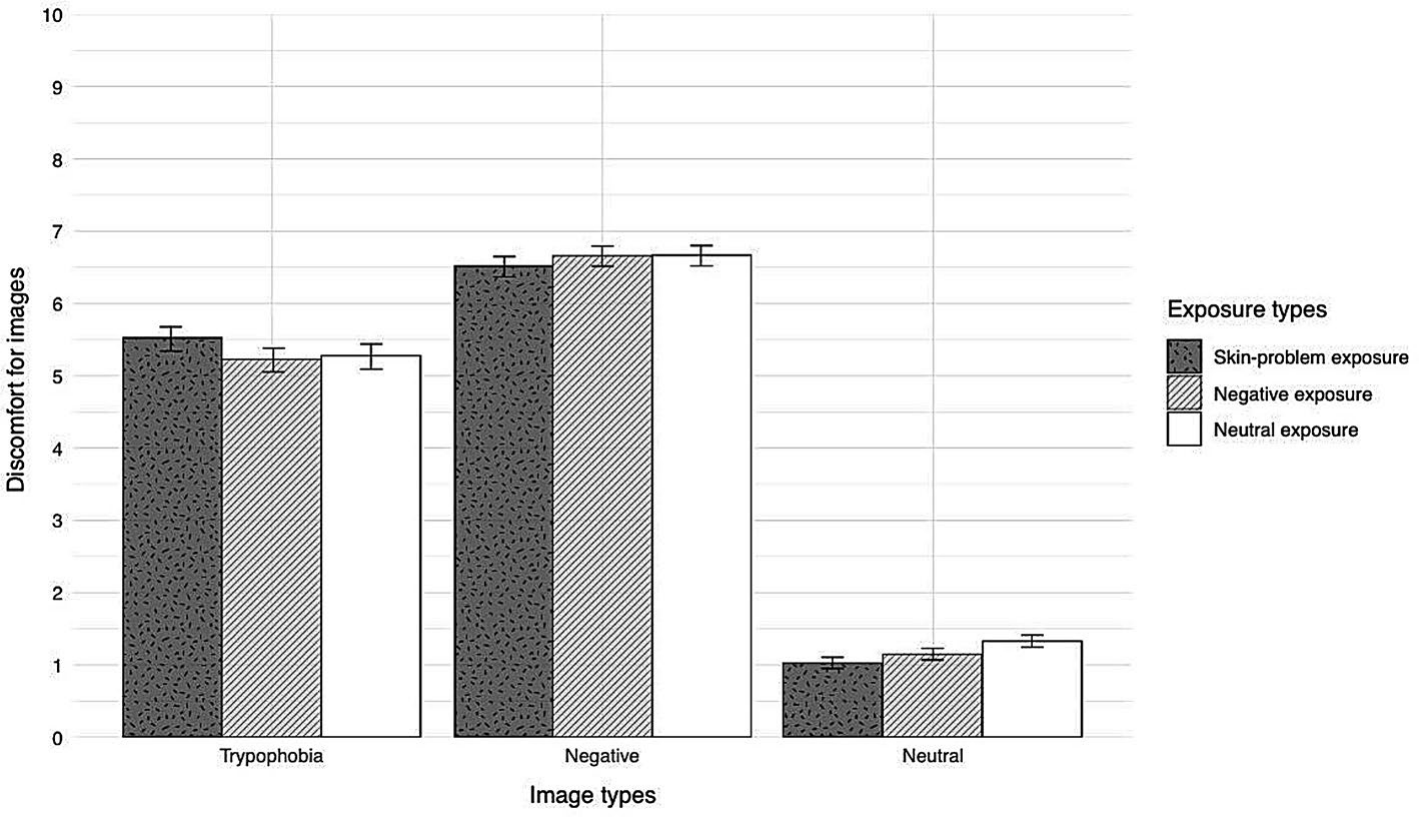
Mean discomfort of images per word type. Error bars indicate the standard error of the mean (*SEM*).

Figure 1 show the mean discomfort of each image type per exposed word type and Table 1 shows the estimated and relative importance values (also see S3).

**Table 1:** Model-based estimates and relative importance values of each parameter.

Parameters		*b*	*SE*	*t*	*p*	
Intercept		4.37	0.05	82.67	< .001	***
Skin-problem word		− 0.06	0.13	− 0.48	.63	
Negative word		− 0.09	0.13	− 0.69	.49	
Trypophobic image		4.16	0.05	92.31	< .001	***
Negative image		5.47	0.05	121.16	< .001	***
Trypophobic image effect	Skin-problem word vs. neutral word	0.56	0.11	5.10	< .001	***
	Negative word vs. neutral word	0.10	0.11	0.92	.36	
Negative image effect	Skin-problem word vs. neutral word	0.16	0.11	1.43	.15	
	Negative word vs. neutral word	0.16	0.11	1.46	.14	

*SE *standard error of the estimate,* Trypophobic image effect *trypophobic image vs. neutral image,* Negative image effect *negative image vs. neutral image*.*

***p < .001.

The results indicated that trypophobic images and negative images predicted an increase in discomfort (*b* = 4.16, *SE* = 0.05, *p* < 0.001; *b* = 5.47, *SE* = 0.05, *p* < 0.001), suggesting that trypophobic and negative images were evaluated as more discomforting than neutral images. Importantly, trypophobic images predicted discomfort in the skin-problem word group (*b* = 0.56, *SE* = 0.11, *p* < 0.001), whereas they did not predict the increased discomfort in the negative word group (*b* = 0.10, *SE* = 0.11, *p* = 0.36). On the other hand, neither skin-problems nor negative words predicted the increased discomfort of negative images (*b* = 0.16, *SE* = 0.11, *p* = 0.15; *b* = 0.16, *SE* = 0.11, *p* = 0.14). Moreover, neither skin-problems nor negative words predicted the discomfort of neutral images (*b* = − 0.06, *SE* = 0.13, *p* = 0.63; *b* = − 0.09, *SE* = 0.13, *p* = 0.49). These results suggested that the discomfort of the trypophobic images increased when participants were exposed to images of skin-related problems.

Experiment 1 was conducted before the COVID-19 (coronavirus disease 2019) outbreak. Since the level of fear of infections and disease is probably different before and after the outbreak, the effects of exposure to skin-problem-related words associated with infection and disease might be different under COVID-19. Therefore, in Experiment 2, we examined the reproducibility of the results of Experiment 1 under the spread of COVID-19 infection. Moreover, the question remains whether the increased negativity for trypophobic images in Experiment 1 was due to exposure to the terms related to skin-problems, potential infections, or both. Therefore, we further examined whether exposure to words associated with infections increased the discomfort caused by trypophobic images, same as the exposure to words associated with skin-problems. Since it was expected that terms related to COVID-19 would be a powerful prime of infection and disease due to the current COVID-19 global pandemic, we tested whether discomfort for trypophobic images was facilitated after exposure to COVID-19 related terms.

## Experiment 2

### Methods

#### Participants

We conducted a power analysis using G*power^29,30^ based on the effect size obtained in Experiment 1 (*d* = 0.21) to determine the minimum sample size required to identify differences in the discomfort of trypophobic images between exposure to skin problems and the neutral condition. It was determined that a minimum of 179 participants per exposure group was required to achieve a power level of 0.90 if we adopted the above effect sizes. Similar to Experiment 1, the participants were recruited on Crowd works. One participant was excluded from the analysis because of problems with properly displaying the images on the screen. Finally, the data of 541 participants (313 women and 228 men; mean age = 40.16 years, SD = 10.96) were collected in Experiment 2. There were 178 participants in the exposure to skin-problems condition (105 women and 73 men; mean age = 40.71 years), 182 in the COVID-19 exposure condition (100 women and 82 men; mean age = 40.02 years), and 181 in the neutral exposure condition (108 women and 73 men; mean age = 39.76 years). Participants provided their informed consent via the Internet before participating in the experiments. Ethical approval for the study was obtained from the Waseda University’s Ethics Review Committee on Research with Human Subjects.

### Stimuli

In addition to the lists of words related to skin-problems and the neutral condition of Experiment 1, we developed and used a new list of words associated with COVID-19. Twenty words related to COVID-19 were selected for the COVID-19 list, including “coronavirus disease” and “close contact” (see S1). We also used the 17 identical trypophobic images that we used in Experiment 1.

### Procedures

The procedures of Experiment 2 were identical to those of Experiment 1, except for those described below.

In Experiment 2, we prepared three types of exposure conditions in the lexical-decision task: the skin-problem exposure, the neutral exposure, and the COVID-19 exposure. The participants evaluated only the discomfort they felt for the trypophobic images. The participants completed the Fear of COVID-19 Scale Japanese version (FCV19SJ ^28^) after completing the lexical-decision task and evaluating the trypophobic images. Participants selected an appropriate number on a 5-point scale ranging from 1 (not true) to 5 (very true) for each of the seven FCV19SJ question items. The total FCV19SJ score was calculated by summing the score for each item.

We planned a one-way analysis of variance (ANOVA) with one between groups factor (skin -problems, COVID-19, and neutral exposure groups). Similar to Experiment 1, we predicted that exposure to skin-problem-related words would increase trypophobic images’ discomfort compared to neutral word exposure. Moreover, we predicted that trypophobic images would be even more discomforting after exposure to COVID-19 associated words than neutral word exposure if the increase in the discomfort of trypophobic images were caused by a fear of general infections rather than a specific fear of skin -problems. We also expected that individuals with higher Fear of COVID-19 Scale scores would be more uncomfortable with trypophobic images.

### Results and discussion

Table 2 shows the negativity for trypophobic images in each exposure condition. We used the nonparametric Kruskal–Wallis test to examine differences in negativity for the trypophobic images in exposure groups because we did not confirm the normality of the score distribution (skin-problems exposure group: W = 0.93, *p* < . 001; COVID-19 exposure group: W = 0.93, *p* < . 001; neutral exposure group: W = 0.95, *p* < . 001). This analysis did not detect any differences among the groups (*χ*^*2*^ (2) = 0.48, *p* = 0.79, η_p_^2^ = 0.0009).

**Table 2:** Negativity of trypophobic images in each exposures group.

Exposure groups	Negativity for trypophobic images	
	Mean	SD
Skin-problems	5.84	2.37
COVID-19	5.96	2.38
Neutral	6.11	1.99

The FCV19SJ scores of the participants in Experiment 2 ranged from 7.00 to 35.00, with a mean score of 18.81. We conducted a Spearman correlation test between FCV19SJ scores and negativity for scores trypophobic images to examine the relationship between the fear of COVID-19 and trypophobia. The results indicated a very weak correlation for all groups (skin-problems exposure: *ρ* = 0.25, *p* < . 001, COVID-19 exposure: *ρ* = 0.26, *p* < . 001, and neutral exposure: *ρ* = 0.25, *p* < . 001), suggesting that the discomfort of trypophobic images might not be significantly related to the fear of COVID-19.

## General discussion

This study demonstrated that trypophobic images were rated as more discomforting than other types of images only when participants were exposed to words related to skin problems, especially in Experiment 1. Crucially, this effect was not observed when the participants were exposed to general negative words. We suggest that exposure to words about skin-related problems resulted in the motivation to avoid contagious skin diseases, which increased the discomfort of trypophobic images. We could not replicate the results of Experiment 1 in Experiment 2. Therefore, we do not emphasize the significance of this effect, and further studies are required in the future. Moreover, the fear of COVID-19 was unrelated to the discomfort of trypophobic images, which implies that the discomfort of trypophobic images is associated with a fear of skin-problems rather than a fear of infections in general.

Importantly, the present study used words referring to completely different physical objects than trypophobic images to activate the concept of skin problems. The results of Experiment 1 are difficult to interpret in terms of visual priming. There are at least two possible explanations of how exposure to words related to skin problems might enhance the discomfort of trypophobic images. First, the implicit activation of semantic, but not visual, representations of contagious skin diseases might facilitate trypophobia. Alternatively, words related to skin problems might direct the observer’s attention (or consciousness) to their body, resulting in enhanced avoidance of contagious skin diseases. The skin-problem words used in this study included not only contagious skin diseases but also non-contagious skin diseases and injuries such as stab-wounds and lacerations. Therefore, the exposure to words related to skin problems when looking at trypophobic images might have directed the observer's attention to his or her body state, resulting in the feeling that the clusters were on his or her body, thereby increasing the discomfort of the trypophobic images. This explanation is consistent with a study that demonstrated extreme discomfort is experienced when clusters are collaged to the self-hand in virtual space^26^. These two possibilities are not mutually exclusive. It is suggested that future studies should examine these possibilities in more detail.

The findings of specific parts of the experiments might also help explain why some people feel a discomfort of repetitive visual patterns. We speculate that the initial discomfort caused by trypophobic images is elicited by the physical properties of trypophobic images, such as the deviation from the 1/*f* structure in the power spectrums^1,6–11^. This discomfort might be modulated by the degree to which an individual implicitly attributes the repetitive visual patterns to negative events such as skin-related problems and/or directs attention to his or her body. Moreover, Sasaki and Yamada^17^ suggested that people with a history of skin diseases evaluated trypophobic images as more discomforting compared to those without a history of such diseases. Therefore, the degree to which repetitive visual patterns are attributed to skin-related problems might be explained by individual characteristics such as having a medical history of skin diseases.

We did not observe any effect of the exposure to skin-problems and COVID-19 on fear of trypophobic images in Experiment 2. The majority of participants were continuously exposed to information about infections and diseases because of the COVID-19 outbreak. Therefore, the priming effect of short exposure to the words of skin-problem and COVID-19 in this experiment might have been attenuated, and the real effects obscured. Future studies must examine if and how COVID-19 has modulated the findings of affective studies and further explore causal relationships between the fear of skin-problem and trypophobia.

In conclusion, the current study demonstrated that exposure to words related to skin diseases promoted the discomfort felt for trypophobic images. Semantic representation of contagiousness and focusing attention on the body might explain the discomfort felt by trypophobic images. However, one limitation of the present study is the small effect size of skin-problem priming on the discomfort for trypophobic images. Nevertheless, these findings provide some, albeit weak, empirical evidence that avoidance from skin diseases is one cause of trypophobia. We suggest that future studies should further explore the causal relationship between activating the concepts of skin-problems and increased discomfort for trypophobic images, which is expected to increase our understanding of the cause of trypophobia.

## Supplementary Information

Supplementary Information.

## Data Availability

The datasets generated during and/or analyzed during the present study are available from the corresponding author on reasonable request.
