# Supplementary material for: Priming with skin-problems increases fear of clusters
Source: Sci Rep. 2021 May 14;11:10362. doi: 10.1038/s41598-021-89917-7 (PMC8121834; doi:10.1038/s41598-021-89917-7)
Supplement: Supplementary file 1 — Supplementary Information. [file 41598_2021_89917_MOESM1_ESM.docx]

Priming with skin-problems increases fear of clusters

*Risako Shirai

Waseda University, Japan

Japan Society for the Promotion of Science, Japan

Hirokazu Ogawa

Kwansei Gakuin University, Japan

Author Note

*Risako Shirai, Faculty of Science and Engineering, University of Waseda, Tokyo, Japan; Japan Society for the Promotion of Science, Tokyo, Japan.

Hirokazu Ogawa, Department of Integrated Psychological Sciences, Kwansei Gakuin University, Japan.

Correspondence concerning this article should be addressed to Risako Shirai, Faculty of Science and Engineering, University of Waseda, Tokyo, Japan. Postal code: 3-4-1 Okubo, Shinjuku-ku, Tokyo, 169-8555, JAPAN. Telephone number: +81 3 5286 3335. E-mail: [RisakoShirai@gmail.com](mailto:RisakoShirai@gmail.com)

**Supplemental materials**

**S1: List of used words and non-words**

| Words | | | | Non-words  (used in Experiments 1 and 2) |
| --- | --- | --- | --- | --- |
| Skin disease and injures  (used in Experiments 1 and 2) | Negative  (used in Experiment 1) | COVID-19  (used in Experiment 2) | Neutral  (used in Experiments 1 and 2) |  |
| 皮膚ガン(skin cancer) | 悪質 (vicious) | 濃厚接触 (close contact) | 観覧車(ferris wheel) | 区華可 |
| 梅毒(syphilis) | 偽装 (impersonation) | 新型コロナ (coronavirus disease) | 泣きぼくろ( mole under one's eye) | 睡いまわし |
| 刺し傷(stab wound) | 違法ダウンロード (illegal download) | せき (cough) | ひよこ(chick) | えみべ |
| 悪性黒色腫(malignant melanoma) | 惨め (miserable) | 呼吸不全 (respiratory failure) | ニュース(news) | ルホエヤ |
| クラミジア(chlamydia) | 愚弄 (taunts) | 発熱 (fever) | いちご(strawberry) | じかぼ |
| ケジラミ(pheasant) | 恥さらし (disgrace) | 倦怠感 (feel excessive fatigue) | 青空(blue sky) | 工飛 |
| 天然痘(smallpox) | 生き恥 (dishonor) | くしゃみ (sneeze) | にわとり(chicken) | しえおい円身 |
| 裂傷(laceration) | 藁人形 (straw man) | 感染 (infection) | 朝日(morning sun) | 求戸 |
| 皮膚潰瘍(skin ulcer) | 破壊 (destruction) | 変異種 (new variant) | 資料庫(storeroom) | 寺己型 |
| わきが(aside) | 浮浪者 (vagrant) | 肺炎 (pneumonia) | 運動(exercise) | おいぷ |
| しらみ(louse) | 背信 (betrayal) | 人工呼吸器 (artificial ventilator) | 環太平洋(pacific rim) | 柳奈呂気 |
| 発疹チフス(typhoid rash) | 自己嫌悪 (self‐hatred) | 陽性 (test positive) | 雨音(sound of rainfall) | 就雷 |
| 尖圭コンジローマ(genital wart) | マイナス思考 (pessimism) | インフルエンザ (influenza) | イソギンチャク(sea anemone) | オリエクジプガ |
| ケロイド(keloidosis) | 賄賂 (bribe) | ウイルス (virus) | プレミアム(premium) | ヒリオデグ |
| 疥癬(scabies) | 憤怒 (rage) | クラスター (epidemic) | デザイン(design) | コヂエヲ |
| 化膿(suppuration) | あさましい (sordid) | 病原体 (pathogen) | エアコン(air conditioner) | デルレペ |
| 帯状疱疹(herpes zoster) | 孤独 (loneliness) | 後遺症 (aftereffect) | 社会(society) | 作河 |
| 凍傷(frostbite) | 高慢ちき (haughty person) | 緊急事態宣言 (state of emergency) | インド料理屋(Indian restaurant) | アルグ図株行 |
| メラノーマ(melanoma) | デメリット (drawback) | マスク (mask) | サイズ(size) | ルレエ |
| 水虫(athlete's foot) | 意気消沈 (despondency) | 感染拡大 (spread of infection) | 公園(park) | 芽禺 |

**S2: Identification numbers of images used**

***Negative images***

OASIS numbers: Gun 8, Knife 1, Police 2, Soldiers 8, War 1, War 3, War 4, War 6, War 7, Fire 7, Shooting 1; IAPS numbers: 9910, 9911, 9912, 9900, 6212, 6838

***Neutral images***

OASIS numbers: Grass 3, Path 1, Skijump 1, Skijump 2, Skyscraper 1, Skyscraper 2, Street 3, Acorns 3, Lake 14, Lake 15, Lake 13, Beach 1, Beach 3, Beach 2, Lake 17, Lake 16, Street 2

**S3: Mean discomfort of each image per word type in Experiment 1**

| Image types | Exposed word types | ***M*** | ***SD*** |
| --- | --- | --- | --- |
| Trypophobic | Skin-problem words | 5.53 | 2.94 |
|  | Negative words | 5.23 | 2.90 |
|  | Neutral words | 5.28 | 2.91 |
| Negative | Skin-problem words | 6.52 | 2.44 |
|  | Negative words | 6.66 | 2.39 |
|  | Neutral words | 6.67 | 2.42 |
| Neutral | Skin-problem words | 1.03 | 1.29 |
|  | Negative words | 1.15 | 1.39 |
|  | Neutral words | 1.33 | 1.51 |


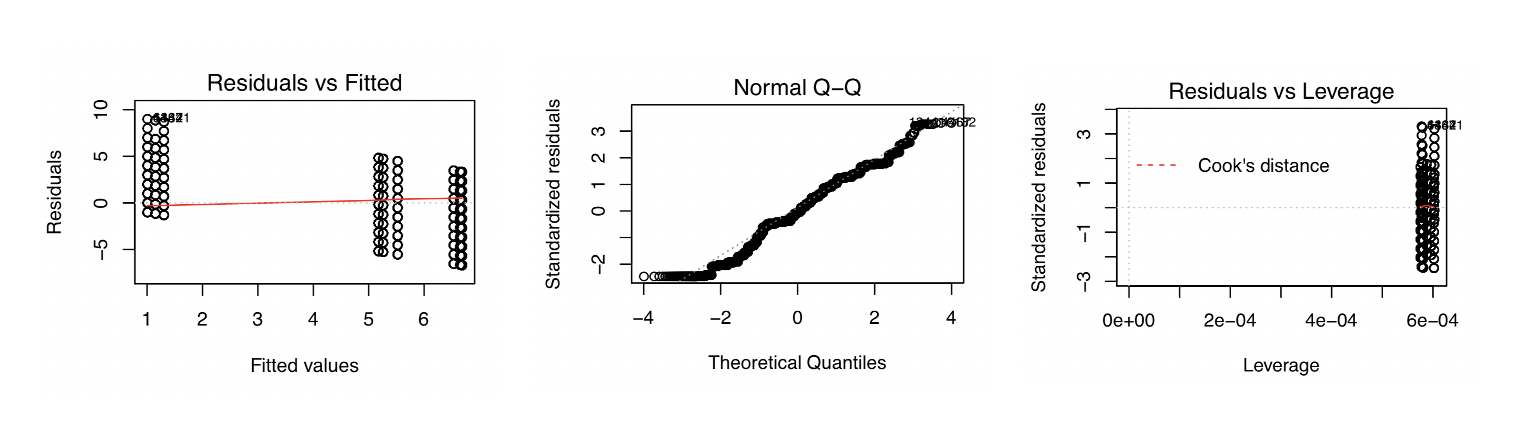
**S4: Model fit and diagnostics information of Experiment 1**

**S5: Analysis by LQMM in Experiment 1**

The discomfort score was also subjected to a linear quantile mixed model (τ = 0.5, number of quadrature knots = 7, type of quadrature = Gauss-Hermite quadrature) with word and image type and their interaction terms via REML. The participants were treated as a random variable. The types of word and image were coded by using contrast coding (word type: reference = “neutral word”; image type: reference = “neutral image”). The linear quantile mixed modelling was conducted by using lqmm packages (Geraci & Bottai, 2014). The following Table shows the estimated and relative importance values.

| Parameters | | ***b*** | ***SE*** | ***Lower bound*** | ***Upper bound*** | ***p*** |  |
| --- | --- | --- | --- | --- | --- | --- | --- |
| Intercept | | 4.48 | 0.08 | 4.32 | 4.64 | < .001 | *** |
| Skin-problem word | | 0.14 | 0.14 | -0.14 | 0.42 | .32 |  |
| Negative word | | -0.04 | 0.12 | -0.27 | 0.19 | .74 |  |
| Trypophobic image | | 4.25 | 0.12 | 4.01 | 4.50 | < .001 | *** |
| Negative image | | 5.74 | 0.17 | 5.41 | 6.07 | < .001 | *** |
| Trypophobic image effect | Skin-problem word *vs.* Neutral word | 0.57 | 0.26 | 0.04 | 1.10 | .04 | * |
|  | Negative word *vs.* Neutral word | 0.01 | 0.25 | -0.49 | 0.51 | .96 |  |
| Negative image effect | Skin-problem word *vs.* Neutral word | 0.09 | 0.23 | -0.37 | 0.55 | .70 |  |
|  | Negative word *vs.* Neutral word | 0.11 | 0.17 | -0.24 | 0.45 | .53 |  |

*Note. SE means the standard error of the estimate. Trypophobic image effect means, “Trypophobic image vs. Neutral image”. Negative image effect means, “Negative image vs. Neutral image”.* *** *p* < .001, * *p* < .05

Geraci, M. & Bottai, M. Linear quantile mixed models. *Statistics and Computing*, **24,** 461–479 (2014).

**S6: One-way ANOVA results of Experiment 2**

| Exposure group | ***F*** | ***p*** | | ***η^2^_p_*** |
| --- | --- | --- | --- | --- |
|  | 0.64 | 0.53 | 0.002 | |
